# Of mice and men: Interaction of *Corynebacterium diphtheriae* strains with murine and human phagocytes

**DOI:** 10.1080/21505594.2019.1614384

**Published:** 2019-05-06

**Authors:** Dulanthi Weerasekera, Tamara Fastner, Roland Lang, Andreas Burkovski, Lisa Ott

**Affiliations:** a Department Biologie, Friedrich-Alexander-Universität Erlangen-Nürnberg, Erlangen, Germany; b Institut für Klinische Mikrobiologie, Immunologie und Hygiene, Universtitätsklinikum Erlangen, Friedrich-Alexander-Universität Erlangen-Nürnberg, Erlangen, Germany

**Keywords:** Cytotoxicity, inflammation, interleukins, macrophages, NFκB, TLRs

## Abstract

Seven non-toxigenic *C. diphtheriae* strains and one toxigenic strain were analyzed with regard to their interaction with murine macrophages (BMM) and human THP-1 macrophage-like cells. Proliferation assays with BMM and THP-1 revealed similar intracellular CFUs for *C. diphtheriae* strains independent of the host cell. Strain ISS4060 showed highest intracellular CFUs, while the toxigenic DSM43989 was almost not detectable. This result was confirmed by TLR 9 reporter assays, showing a low signal for DSM43989, indicating that the bacteria are not endocytosed. In contrast, the non-pathogenic *C. glutamicum* showed almost no intracellular CFUs independent of the host cell, but was recognized by TLR9, indicating that the bacteria were degraded immediately after endocytosis. In terms of G-CSF and IL-6 production, no significant differences between BMM and THP-1 were observed. G-CSF production was considerably higher than IL-6 for all *C. diphtheriae* strains and the *C. glutamicum* did not induce high cytokine secretion in general. Furthermore, all corynebacteria investigated in this study were able to induce NFκB signaling but only viable *C. diphtheriae* strains were able to cause host cell damage, whereas *C. glutamicum* did not. The absence of Mincle resulted in reduced G-CSF production, while no influence on the uptake of the bacteria was observed. In contrast, when MyD88 was absent, both the uptake of the bacteria and cytokine production were blocked. Consequently, phagocytosis only occurs when the TLR/MyD88 pathway is functional, which was also supported by showing that all corynebacteria used in this study interact with human TLR2.

## Introduction

*Corynebacterium diphtheriae* is the classical etiological agent of diphtheria and the type species of the genus *Corynebacterium* [1,2]. The transmission of *C. diphtheriae* from person to person occurs by close physical contact or respiratory droplets [3]. Infections of the upper respiratory tract are characterized by sore throat, low fever, and malaise. Symptoms range from mild pharyngitis to severe hypoxia with pseudomembrane formation due to the toxin. Vaccination against classical respiratory diphtheria is available with toxoid vaccine that is directed against the toxin. Nevertheless, *C. diphtheriae* does not mandatorily produce the diphtheria toxin; it has to be infected by a toxin carrying *β*-corynephage, integrating into the genome of the bacteria [4]. Interestingly, toxigenic but also non-toxigenic *C. diphtheriae* strains are increasingly associated with invasive infections, such as endocarditis, osteomyelitis, splenic abscesses, meningitis, and septic arthritis [5–7]. Recent characterization of a non-toxigenic *C. diphtheriae* strain isolated from a cancer patient with osteomyelitis indicates that *C. diphtheriae* can colonize not only epithelia but can also infect deeper parts of the body [8].

Besides the mode of action of the diphtheria toxin, the molecular mechanisms of the interaction of *C. diphtheriae* with host cells, especially the activation of human macrophages by non-toxigenic strains, is poorly understood. In case of mycobacteria, which are closely related to corynebacteria, the molecular mechanisms of the infection process are investigated in more detail. For the human pathogen *Mycobacterium tuberculosis* it is known that direct recognition of trehalose dimycolate (TDM), the major lipid in the outer membrane of mycobacteria, by the C-type lectin receptor (CLR) Mincle triggers macrophage activation through the adaptor protein Fc receptor gamma chain (FcRץ), the kinase Syk and the Card9-Bcl10-Malt1 complex [9–11]. Mycobacteria possess a number of Toll-like receptor (TLR) ligands, e.g. the 19 kDa lipopeptide and lipoarabinomannans. The activation of the TLR signaling pathway originates from the cytoplasmic Toll/IL-1 receptor (TIR) domain that associates with a TIR domain-containing adaptor, MyD88. MyD88 links IL-1 receptor (IL-1R) or TLR family members to IL-1R-associated kinase (IRAK) family kinases via homotypic protein–protein interaction. Activation of IRAK family kinases leads to a variety of functional outputs, including the activation of nuclear factor-kappa B (NFκB) making MyD88 a central node of inflammatory pathways [12].

In case of *C. diphtheriae*, it is known that macrophages show a delay of phagolysosome formation when being infected with different *C. diphtheriae* strains [13]. Additionally, cell wall extracts of *C. diphtheriae* play a role in activating murine macrophages [14]. Furthermore, TLR2 was found to be required for the upregulation of Mincle expression upon corynebacterial infection [14]. To get deeper insights into the molecular mechanisms of macrophage activation by *C. diphtheriae*, we investigated the interaction of *C. diphtheriae* wild type strains with bone marrow-derived macrophages (BMM) in comparison to the human THP-1 cell line. By using a combination of BMM, Mincle^−/-^ and MyD88^−/-^ cells, we tested if viable *C. diphtheriae* strains bind and activate the C-type lectin receptor Mincle and the Toll-like receptor TLR2. Furthermore, the role of the adaptor protein Myd88 during uptake of the bacteria was investigated. By quantification of intracellular bacteria, detection of pro-inflammatory cytokines, analysis of NFκB activation as well as host cell damage, host immune response was studied during *C. diphtheriae* infection. The question of recognition of bacteria in endolysosomal compartments was addressed by using TLR9 reporter cells.

## Materials and methods

### Bacterial strains and growth conditions

*C. diphtheriae* strains as well as *C. glutamicum* (Table 1) were grown in Heart Infusion (HI) broth (Becton Dickinson, Sparks, MD, USA) or on HI and Brain Heart Infusion (BHI) (Oxoid, Wesel, Germany) agar plates as well as Columbia Blood Agar (CBA) containing 5% sheep blood (Oxoid, Wesel, Germany) at 37°C.

**Table 1.** Bacterial strains and cell lines used in this study.

	Genotype/Description	Reference/Source
***C. diphtheriae*strains**		
ISS3319	*C. diphtheriae* var. mitis, non-toxigenic, isolated from patients affected by pharyngitis/tonsilitis	[29]
ISS4060	*C. diphtheriae* var. gravis, non-toxigenic, isolated from patients affected by pharyngitis/tonsilitis	[29]
ISS4746	*C. diphtheriae* var. gravis, non-toxigenic, isolated from patients affected by pharyngitis/tonsilitis	[6]
ISS4749	*C. diphtheriae* var. gravis, non-toxigenic, isolated from patients affected by pharyngitis/tonsilitis	[6]
DSM43988	strain 48,255, ATCC 11,913, avirulent throat culture	obtained from DSMZ, Braunschweig, Germany
DSM43989	PW strain, strain 5159, ATCC 13,812, producer of diphtheria toxin for toxoid production	[30], obtained from DSMZ, Braunschweig, Germany
DSM44123	type strain, C7s, ATCC 27,010, CIP 100,721, NCTC 11,397	obtained from DSMZ, Braunschweig, Germany
Inca-402	isolated from a bronchial wash specimen from a cancer patient with pneumonia in Rio de Janeiro, 2000	[31]
NCTC13129	biovar gravis; *tox^+^*	[32]
***C. glutamicum* strains**		
ATCC 13032	type strain	[33], laboratory stock
**Cell lines**		
THP-1	human leukemic monocytic cells	[34]
THP-1-Blue NFκB	THP-1 cells with stable integrated NFκB inducible SEAP reporter construct	InvivoGen, San Diego, USA
HEK-Blue 293 hTLR2	human TLR2/NF-κB/SEAP reporter HEK293 cells	InvivoGen, San Diego, USA
HEK-Blue 293 hTLR9	human TLR9/NF-κB/SEAP reporter HEK293 cells	InvivoGen, San Diego, USA
**Bone marrow-derived macrophages**		
C57/BL6	bone marrow cells from femur and tibiae differentiated to macrophages	Präklinisches Experimentelles Tierzentrum of the Medical Faculty of the Friedrich-Alexander Universtity Erlangen-Nuremberg
Clec4e^−/-^	bone marrow cells from femur and tibiae of Mincle-deficient mice differentiated to macrophages	Präklinisches Experimentelles Tierzentrum of the Medical Faculty of the Friedrich-Alexander Universtity Erlangen- Nuremberg
MyD88^−/-^	bone marrow cells from femur and tibiae of Myd88-deficient mice differentiated to macrophages	Präklinisches Experimentelles Tierzentrum of the Medical Faculty of the Friedrich-Alexander Universtity Erlangen- Nuremberg

### Isolation and culture of primary mouse macrophages and macrophage cell lines

Killing of mice by cervical dislocation for extraction of bone marrow from femural and tibial bones was registered according to German Animal Protection Law §4 with the Regierung von Mittelfranken (protocol number 12/08). C57BL/6, Clec4e^−/-^ and MyD88^−/-^ mice were bred at the Präklinisches Experimentelles Tierzentrum of the Medical Faculty of the Friedrich-Alexander University Erlangen-Nürnberg. Bone marrow cells from femurs and tibiae were differentiated to macrophages by culture in complete Dulbecco’s modified Eagle’s medium (DMEM) (Life Technologies, Carlsbad, USA) containing 100 U penicillin ml^−1^ and 0.1 mg streptomycin ml^−1^, 10 % fetal calf serum (FCS) (Gibco BRL, Germany) and 50 µM *β*-mercaptoethanol (complete DMEM [cDMEM]) plus 10% L929 cell-conditioned medium as a source of macrophage colony-stimulating factor (M-CSF), as previously described [15]. On day 7, adherent macrophages were harvested by Accutase (Sigma, Munich, Germany) treatment, washed with 1 x PBS and counted.

THP-1 human monocytes were cultured in 10% FCS supplemented RPMI medium 1640 (Gibco BRL, Eggenstein, Germany) (containing 100 U penicillin ml^−1^ and 0.1 mg streptomycin ml^−1^) at 37°C in 5% CO_2_ in a humidified cell culture incubator. The cells were harvested by centrifugation, washed with 1 x PBS and counted.

### Replication assay

For gentamicin protection assays, cells were seeded in 24-well plates (Nunc, Wiesbaden, Germany) at a density of 2 × 10^5^ cells per well 24 h prior to infection. In case of THP-1 cells, 10 ng ml^−1^ phorbol 12-myristate 13-acetate (PMA) were added for differentiation of the cells. Overnight cultures of *C. diphtheriae* grown in HI were re-inoculated to an OD_600_ of 0.1 in fresh medium and grown to an OD_600_ of 0.4 to 0.6. An inoculum with MOI of 1 or 10 was prepared in RPMI without antibiotics and 500 µl per well were used to infect the cells. Serial dilutions of the inoculum were plated on blood agar plates (Oxoid, Wesel, Germany) using an Eddy Jet Version 1.22 (IUL Instruments, Barcelona, Spain) and incubated at 37°C for 2 days. The infection plates were centrifuged for 5 min at 350 x g to synchronize the infection and incubated for 30 min (37°C, 5% CO_2_, 95% humidity) to allow phagocytosis of bacteria. Subsequently, the supernatant containing non-engulfed bacteria was aspirated, cells were washed once with PBS and remaining extracellular bacteria were killed by addition of 100 mg ml^−1^ gentamicin in cell culture medium. After 2 h, cells were either lysed by adding 500 µl of 0.1% Triton X-100 in PBS and intracellular bacteria were recovered by plating serial dilutions of the lysates on blood agar plates or further incubated with medium containing 10 mg ml^−1^ gentamicin for analysis at later time points (4 and 20 h). After incubation at 37°C for 2 days, the number of colony forming units (CFU) was determined. The ratio of bacteria used for infection (number of colonies on inoculum plates) and bacteria in the lysate (number of colonies on the lysate plates) multiplied with 100 gave the percentage of viable intracellular bacteria at different time points. When the survival of intracellular bacteria in THP-1 cells was analyzed over the time, the number of CFU at 2 h was set to 100% and later time points were calculated based on this value. The assay was performed at least in three biological replicates each performed in triplicates and means and standard deviations were calculated.

### NFκB reporter assay

THP1-Blue NFκB cells (InvivoGen, San Diego, USA) carrying a stable integrated NFκB-inducible secreted embryonic alkaline phosphatase (SEAP) reporter construct were used to analyze NFκB induction by *C. diphtheriae. C. diphtheriae* strains were inoculated from an overnight culture to an OD_600_ of 0.1 in fresh medium and grown to an OD_600_ of 0.4 to 0.6. An inoculum with an OD_600_ of 1.25 in 1,000 µl PBS was prepared and 20 µl of this inoculum or of the 10^−1^ and 10^−2^ dilutions were mixed with 180 µl of a suspension with 5 × 10^5^ THP1-Blue NFκB cells in cell culture medium resulting in an MOI of 100, 10 or 1. UV-killed bacteria in the same concentrations were also studied. After incubation for 20 h at cell culture conditions, the 96-well plates were centrifuged (350 x g, 5 min) and 20 µl of the cell free supernatant was mixed with 180 µl pre-warmed SEAP detection reagent QUANTI-Blue (InvivoGen, San Diego, USA). After further incubation at cell culture conditions for 3 h, the levels of NFκB-induced SEAP resulting in a color change from pink to blue were measured in a microplate reader (TECAN Infinite 200 PRO, Männedorf, Switzerland) at 620 nm.

### Determination of cytokine excretion

For determination of cytokine activation through *C. diphtheriae*, supernatants of infected cells were collected after different time points and stored at −20°C. IL-6 and G-CSF concentrations were measured using the DuoSet ELISA Kits according to the manufacturer’s recommendations (R&D systems). Briefly, the ELISA plates were coated over night with a capture antibody at room temperature, washed 3 times with 0.05% Tween20 in PBS, blocked for 1 h at room temperature with 1% BSA in PBS and washed again 3 times. Subsequently, 100 µl supernatant of infected cells or standard dilutions were added, and the plates were incubated for 2 h, washed again three times and further incubated for 2 h at room temperature. After another washing step, a streptavidin-HRP solution was added and the plates were stored for 20 min under light exclusion, washed again, and incubated for another 20 min in the dark with substrate solution. To stop the color reaction, 2 N H_2_SO_4_ was added to the wells and the optical density was determined using a microplate reader (TECAN Infinite 200 PRO, Männedorf, Switzerland) set to 450 nm with wavelength correction at 550 nm.

### LDH release

The release of cytosolic lactate dehydrogenase (LDH) as a sign of host cell damage during infection was measured using the cytotoxicity detection kit according to the supplier (Roche).

Briefly, 100 µl supernatant of infected cells were mixed with 2.5 ml of the provided catalyst solution and 112.5 µl of the provided dye solution in 96-well plates, incubated in the dark for 30 min and the absorbance was measured at 490 nm and wavelength correction at 620 nm in a microplate reader (TECAN Infinite 200 PRO, Männedorf, Switzerland). Cells treated with 2% Triton X-100 served as positive control for maximal LDH release and were set to 100%, untreated cells served as negative control.

### Griess assay

NO production was assessed via a Griess assay (Griess Reagent System, Promega). 50µl of the culture medium supernatant were gently mixed with an equal volume of sulfanilamide solution and incubated in the dark at room temperature for 10 min. Subsequently, 50 µl of NED solution was added and the reaction solution was incubated in the dark at room temperature for 10 min. The absorbance of this solution at 540 nm was measured in a microplate reader (TECAN Infinite 200 PRO, Männedorf, Switzerland) and the nitrite concentration was calculated from a nitrite standard reference curve.

### TLR2 and TLR9 reporter assays

HEK-Blue-hTLR2 and hTLR9 cells (InvivoGen, San Diego, USA), carrying a stable integrated inducible secreted embryonic alkaline phosphatase (SEAP) reporter construct, were used to analyze TLR2 and TLR9 binding of *C. diphtheriae*. Stimulation with a TLR2 or TLR9 ligand activates NF-κB and AP-1, which induce the production of SEAP. TRL2 is a surface exposed receptor, while TLR9 is expressed in the endoplasmic reticulum and located in endolysosomal compartments, where it recognizes specific unmethylated CpG sequences, which are characteristic for bacterial DNA. Furthermore, TLR9 binding involves Myd88 signaling. *C. diphtheriae* strains were inoculated from an overnight culture to an OD_600_ of 0.1 in fresh medium and grown to an OD_600_ of 0.4 to 0.6. An inoculum with an OD_600_ of 1 in 1000 µl PBS was prepared and 20 µl of the 10^−1^ and 10^−2^ dilutions of this inoculum for hTLR2 and 1:1 and 1:10 dilutions for hTLR9 were mixed with 180 µl of a suspension with 5 × 10^5^ HEK-Blue hTRL2 in cell culture medium or hTLR9 cells in HEK Blue detection medium. This resulted in an MOI of 10 or 1 for hTLR2 and an MOI 50 and 10 for hTLR9. UV-killed bacteria in the same amount were also investigated. After incubation of HEK-Blue hTLR2 cells for 20 h at cell culture conditions, 96-well plates were centrifuged (350 x g, 5 min) and 20 µl of the cell-free supernatant was mixed with 180 µl pre-warmed SEAP detection reagent Quanti-Blue (InvioGen, San Diego, USA). After further incubation at cell culture conditions for 3 h, SEAP activity was measured in a microplate reader (TECAN Infinite 200 PRO, Männedorf, Switzerland) at 620 nm. In contrast, HEK-Blue hTLR9 cells were incubated for 24 h under cell culture conditions and SEAP activity was measured as mentioned above.

### Statistical analysis

All experiments were carried out in at least three independent biological replicates, each performed in technical triplicates and standard deviations were calculated. Data were analyzed using the software GraphPad Prism 7.0 (GraphPad, CA, USA).

## Results

### *Survival of* C. diphtheriae *after internalization by primary macrophages from C57BL/6 mice, Clec4e- and Myd88-deficient mice*

**Figure 1.**
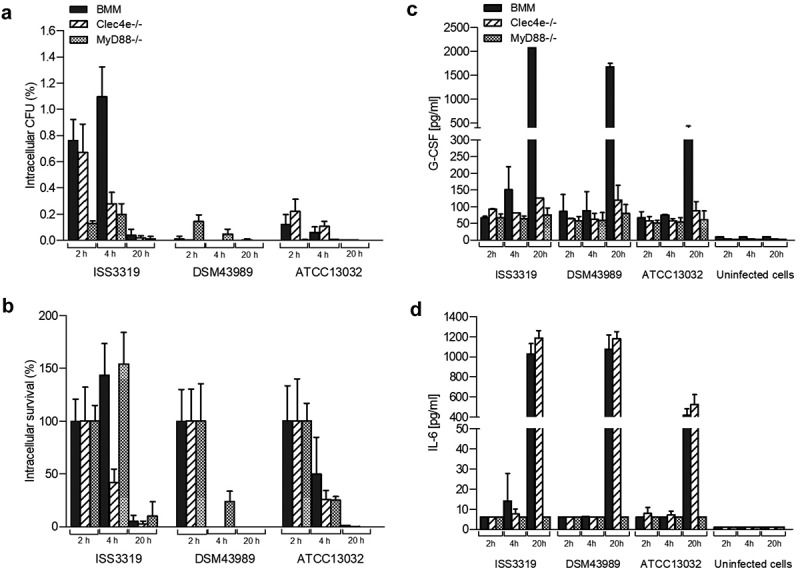
Quantitative analysis of viable intracellular corynebacteria and cytokine ELISA of bone marrow-derived murine macrophages from C57/BL6 (BMM), Clec4e- and MyD88-deficient mice after infection with bacteria. Cells were infected with *C. diphtheriae* wild type strains ISS3319, DSM43989 and *C. glutamicum* ATCC 13032 at an MOI of 10 for 2, 4 and 20 h, lysed and the lysates were plated on blood agar plates. (a) Intracellular CFU in percent of the inoculum. (b) intracellular survival in percent of the bacteria that were taken up after 2 h. Supernatants of primary bone marrow (BMM), Clec4e^−/-^ and MyD88^−/-^ macrophages infected with bacteria were collected at different time points post-infection (2, 4 and 20 h) and used for the determination of (c) G-CSF and (d) IL-6 concentrations. Data shown are mean values of at least three independent biological replicates each performed in triplicates ± standard deviation.

As a basis for subsequent experiments, *C. diphtheriae* interaction with bone marrow-derived murine macrophages (BMM), Clec4e- and Myd88-deficient cells was analyzed (Figure 1). Different *C. diphtheriae* wild type isolates and the non-pathogenic *C. glutamicum* strain ATCC 13032 were studied in respect to internalization (Figure 1(a)) and intracellular survival (Figure 1(b)). When the intracellular CFUs were analyzed, strain-specific and cell-specific differences were detectable. Almost no viable bacteria of the non-pathogenic *C. glutamicum* strain (ATCC 13032) and the toxigenic *C. diphtheriae* strain (DSM43989) were observed already after 2 h post-infection independent of the cell type (Figure 1(a)). For the other investigated strains, the CFUs after 2 h ranged between 0.5% and 1.5% of the inoculum in BMM, which is representatively shown for strain ISS3319. The 2 h values were set to 100% and the survival rates were calculated (Figure 1(b)). The calculated rates at 2 and 4 h showed, that strain ISS3319 is able to proliferate within the first 4 h post-infection in BMM and MyD88^−/-^, while the survival rates of other *C. diphtheriae* strains, except the toxigenic strain DSM43989, remained at least constant. All strains were almost completely degraded overnight (Figure 1(b)). All other *C. diphtheriae* strains behaved similar to ISS3319 and the results for all eight strains are shown in the supplementary material (Fig. S1, S2, S3).

In summary, the different non-toxigenic *C. diphtheriae* isolates were able to persist longer periods within the BMM compared to *C. glutamicum* and toxigenic DSM43989, and were partially able to proliferate within the first 4 h post-infection. This result indicated that *C. diphtheriae* is able to cause a delay of phagolysosome maturation in macrophages, which was shown in a previous study for different *C. diphtheriae* strains [13].

### *Role of the C-type lectin receptor mincle on* C. diphtheriae *infection*

In order to address if macrophage activation via Mincle is influenced when viable bacteria are used for infection, BMM from Mincle-deficient mice (Clec4e^−/-^) were incubated with *C. diphtheriae* (Figure 1). These experiments revealed internalization rates between 0.6 and 1.0% for all *C. diphtheriae* strains, except the toxigenic strain DSM43989. Compared to the infection of BMM of wild type C57BL/6 mice, there was no significant reduction of the detectable CFUs in Clec4e^−/-^ mice. As observed in infection of BMM wild type cells, in case of the toxigenic strain DSM43989 almost no viable intracellular CFUs were detectable already after 2 h of infection and the number of intracellular CFUs of *C. glutamicum* ATCC 13032 was significantly lower compared to the non-toxigenic *C. diphtheriae* strains, such as ISS3319 (Figure 1(a)). The survival rates reached about 30 to 60% of those of the non-toxigenic *C. diphtheriae* strains. Also *C. glutamicum* were still viable within the first 4 h post-infection. For strain DSM43989, no colony was detectable 4 h post-infection. After overnight incubation, the strains ISS4060, DSM44123 and Inca-402 showed the highest resistance to macrophage action with about 10 to 15% of the internalized bacteria being still viable (Fig. S2). The non-pathogenic control strain ATCC 13032 was almost entirely degraded overnight. Noteworthy, by comparing the intracellular CFUs in Clec4e^−/-^ BMM to C57BL/6 BMM no significant difference was observed.

### *Influence of the adaptor protein Myd88 on* C. diphtheriae *infection*

The Toll-like receptor/MyD88 pathway, a major pattern recognition system, plays an important role in immunity. The MyD88 adaptor molecule is recruited after activation of most TLRs. This pathway includes the recruitment and activation of several other proteins and leads finally to the activation of NFκB pathways and the production of pro-inflammatory cytokines [16,17]. In this study, the role of the MyD88 adapter protein in the activation of macrophages by viable *C. diphtheriae* strains in comparison to BMM and Clec4e^−/-^ was analyzed (Figure 1). Replication assays carried out with cells derived from MyD88-deficient mice revealed that after 2 h of infection intracellular CFUs of maximum 0.3% of the inoculum were detectable for all *C. diphtheriae* strains and the number of intracellular CFUs remained almost constant within 4 h post-infection. Less than 0.01% of the inoculum was observed for strain *C. glutamicum* ATCC 13032 (Figure 1(a)). All *C. diphtheriae* strains, except the toxigenic one (DSM43989), were able to proliferate, or the number of viable bacteria remained constant at least the first 4 h post-infection. After overnight incubation, almost no CFUs were detectable for each strain (Figure 1(b)). Comparison the internalization rates of the different strains in MyD88-deficient cells with those in wild type cells after 2 h showed a reduction by 80 to 90% of the intracellular CFUs. The data indicated that the recruitment of the adaptor protein MyD88 is essential for TLR signaling in macrophage activation by viable *C. diphtheriae* strains.

### *Response of primary macrophages to* C. diphtheriae *infection*

In order to address the immune response to the infection with *C. diphtheriae* in BMM, Clec4e- and Myd88-deficient cells, the supernatants were collected at 2, 4 and 20 h post-infection for determination of cytokine secretion, such as G-CSF (Figure 1(c)) and IL-6 (Figure 1(d)), respectively. Analysis of cytokines in the supernatants from BMM infected with non-toxigenic *C. diphtheriae* strains revealed about 2000 pg ml^−1^ of G-CSF after overnight incubation, which is shown representatively for strain ISS3319 (Figure 1(c)). The toxigenic strain DSM43989 reached slightly lower values of about 1700 pg ml^−1^ of G-CSF, while the non-pathogenic control strain ATCC 13032 reached only about 300 pg ml^−1^ of G-CSF (Figure 1(c)). Interestingly, the weak cytokine production in response to *C. glutamicum* infection may not be due to the low intracellular CFUs that were detected in the proliferation assay for this strain. Rather it seems to indicate the non-pathogenic role of strain ATCC 13032, since the intracellular CFUs of strain DSM43989 were even lower than those of *C. glutamicum*, but were leading to high G-CSF production (Figure 1(a)). In Mincle-deficient cells, almost no G-CSF production was observed at 2 and 4 h post-infection, which was comparable to the wild type cells. Interestingly, after overnight incubation, the G-CSF concentrations were also almost at background levels between 100 and 270 pg ml^−1^. Compared to macrophages derived from C57BL/6 mice, this is a reduction of about 90% (Figure 1(c)). The measurement of the G-CSF concentrations in the supernatant of Myd88-deficient cells revealed a reduction to background level at all time points (Figure 1(c)).

When IL-6 concentrations were measured in the supernatant of the different cell types, no significant differences were observed between BMM and Clec4e^−/-^ cells (Figure 1(d)). Interestingly, after overnight incubation all *C. diphtheriae* strains lead to strong IL-6 production in both BMM and Clec4e^−/-^ cells. The non-pathogenic strain ATCC 13032 reached the lowest IL-6 levels of about 400 pg ml^−1^ (Figure 1(d), S1). IL-6 concentrations in Mincle-deficient cells in response to *C. diphtheriae* infection reached values between 1000 and 1200 pg ml^−1^ for all *C. diphtheriae* strains, which was almost similar to wild type cells. Again, the lowest value of about 600 pg ml^−1^ was observed for strain ATCC 13032 (Figure 1(d)). When IL-6 secretion was analyzed in Myd88-deficient cells, all strains reached values only to background level of 6.15 pg ml^−1^ at all time-points (Figure 1(d)).

In summary, infection of BMM with *C. diphtheriae* induced production of the inflammatory cytokines G-CSF and IL-6 (Figure 1(c,d)). The non-pathogenic strain ATCC 13032 caused significantly lower inflammatory response compared to pathogenic corynebacteria. Furthermore, Mincle-binding of pathogenic corynebacteria seems not mandatory for the internalization of the bacteria. However, the inflammatory response in terms of G-CSF, but not of IL-6, is impaired when Mincle is absent. When the adaptor protein MyD88 is absent in macrophages neither phagocytosis of the bacteria nor G-CSF or IL-6 release occurred.

### *Survival of* C. diphtheriae *after internalization by THP-1 cells*

**Figure 2.**
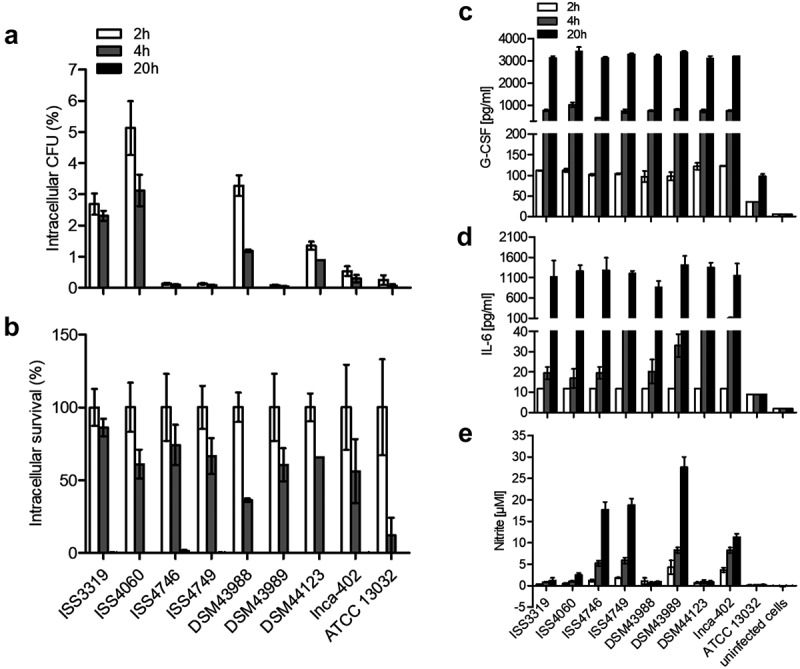
Quantitative analysis of viable intracellular corynebacteria in THP-1 cells and evaluation of cytokine and nitric oxide production. THP-1 cells were infected with different *C. diphtheriae* wild type strains and *C. glutamicum* ATCC 13032 at an MOI of 10 for 2, 4 and 20 h, lysed and the lysates were plated on blood agar plates. (a) Intracellular CFU in percent of the inoculum. (b) Intracellular survival in percent of the bacteria taken up after 2 h. Supernatants of THP-1 cells infected with bacteria were collected at different time points post-infection (2, 4 and 20 h) and used as samples for determination of (c) G-CSF and (d) IL-6 and (e) NO concentrations. Data shown are mean values of at least three independent biological replicates each performed in triplicates ± standard deviation.

While murine cells are an excellent model to test the influence of several signaling pathways due to the availability of the respective knockout cells, humans are the natural host of *C. diphtheriae*. Therefore, the human macrophage-like cell line THP-1 was applied to analyze internalization and survival of *C. diphtheriae* (Figure 2). In this case, strong strain-specific differences in the uptake of the bacteria were observed. After 2 h of incubation, the detected intracellular CFUs of the strains ISS4746, ISS4749 and the toxigenic strain DSM43989 were less than 0.2% of the inoculum. Highest internalization rates were reached for strains ISS3319 (~2.5%), ISS4060 (~5.0%) and strain DSM43988 (~3.0%) while the lowest internalization rates were observed for strains DSM44123 and Inca-402 with about 1.3% and 0.5%, respectively. The non-pathogenic strain ATCC 13032 was almost not detectable already 2 h post-infection (Figure 2(a)). By calculation of the survival rates, it became apparent that all *C. diphtheriae* strains were able to persist within the cells at least up to 4 h post-infection, but were completely degraded overnight (Figure 2(b)).

### *Response of THP-1 cells to* C. diphtheriae *infection*

To investigate the reaction of unstimulated THP-1 cells in response to *C. diphtheriae* infection, culture supernatants were collected at 2, 4 and 20 h post-infection to measure G-CSF (Figure 2(c)), IL-6 (Figure 2(d)) and NO production (Figure 2(e)). Interestingly, in contrast to the strain-specific internalization of the bacteria into the cells, no significant differences in cytokine-production where observed (Figure 2(c,d)). After 4 h of infection *C. diphtheriae* strains led to G-CSF secretion of about 750 pg ml^−1^ (Figure 2(c)), while no IL-6 production was measurable (Figure 2(d)). After overnight, values of more than 3000 pg ml^−1^ were reached for G-CSF production (Figure 2(c)) and values between 800 and 1400 pg ml^−1^ were detectable for IL-6 (Figure 2(d)).

In THP-1 cells, reactive nitrogen species contributed to innate host defense against several *C. diphtheriae* wild type strains (Figure 2(e)). 17 µM nitrite for strains ISS4746 and IS4749, 27 µM nitrite for strain DSM43989 and 10 µM nitrite for strain Inca-402 at 20 h post-infection were detected. Remarkably, the NO production was contrary to the viable CFUs that were detectable at 2 h post-infection (Figure 2(a)), meaning that the strains that showed low numbers of intracellular CFUs induced high NO production in THP-1 cells. The high NO levels in response to some strains may result in fast degradation or inactivation of the bacteria and therefore no CFUs were detectable at 2 h post-infection. Considering this result, the 2 h values may not mandatory represent the uptake of the bacteria or the phagocytosis rate; most of the bacteria could already be killed by reactive nitrogen species within the first 2 h of infection in a strain-specific manner. Furthermore, it is conceivable that the high NO production leads to cell death of the macrophage [18].

**Figure 3.**
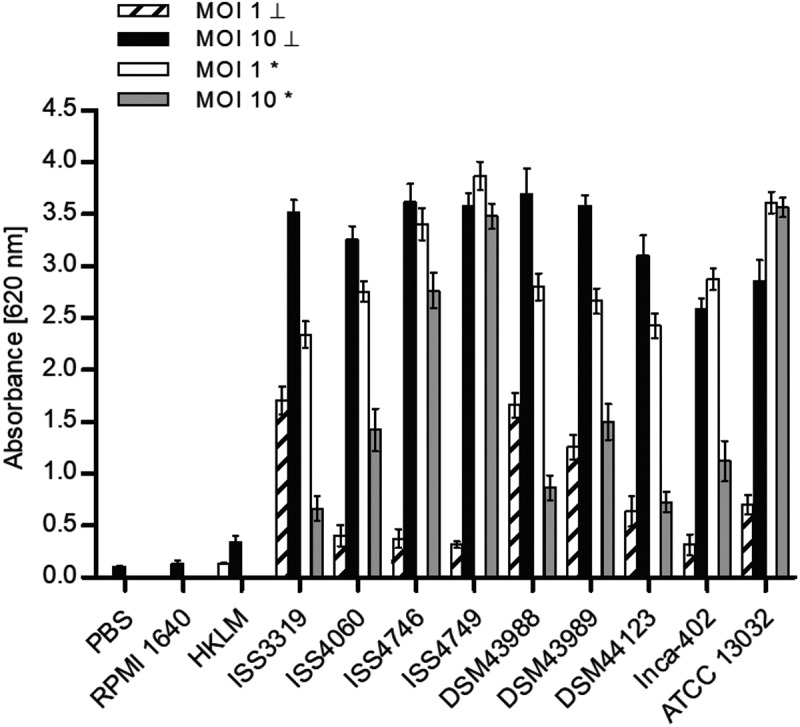
NF-KB activation in response to *C. diphtheriae* infection. THP1-Blue NF-KB cells were incubated for 20 h with viable and UV-killed bacteria of the non-pathogenic *C. glutamicum* ATCC 13032 and pathogenic *C. diphtheriae* strains at an MOI 1 and 10, respectively. Subsequently, supernatants were taken and mixed with QuantiBlue SEAP detection solution leading to a change in color upon NF-KB activation, which was detected by measuring absorbance at 620 nm. Dead bacteria are indicated with (┴) and live bacteria with (*). Data shown are mean values of three independent biological replicates each performed in triplicates ± standard deviation.

When NFκB induction was analyzed in response to *C. diphtheriae* infection, cells of the reporter cell line THP-1-Blue NFκB were incubated for 20 h with MOI 1 and 10 of UV-killed and viable bacteria, respectively (Figure 3). Using MOI 1 and 10 of dead *C. diphtheriae* stains resulted in dose-dependent NFκB activation. The non-pathogenic strain ATCC 13032 led to similar NFκB signals independent of the MOI. In contrast, when viable *C. diphtheriae* strains were tested, using MOI 1 led to significant higher NFκB activation compared to MOI 10. A reduced NFκB signal up to 70% at MOI 10 (dead bacteria) was observed. This may indicate a specific reaction or detrimental effects of *C. diphtheriae* to the cells. Furthermore, strain DSM43989, which was shows very low numbers of intracellular CFUs at 2 h of infection (Figure 2(a)) and led to high NO production after 20 h (Figure 2(e)) showed a strong reduction of NFκB activation when MOI 10 of viable bacteria was used compared to MOI 1 (Figure 3). This observation may be explained by the fact that this strain is the only diphtheria toxin-producing strain used in this assay and THP-1 cells are expressing the corresponding receptor HB-EGF (heparin-binding EGF-like growth factor), which allows entrance of the toxin, leading to blocking of protein synthesis in the cell.

**Figure 4.**
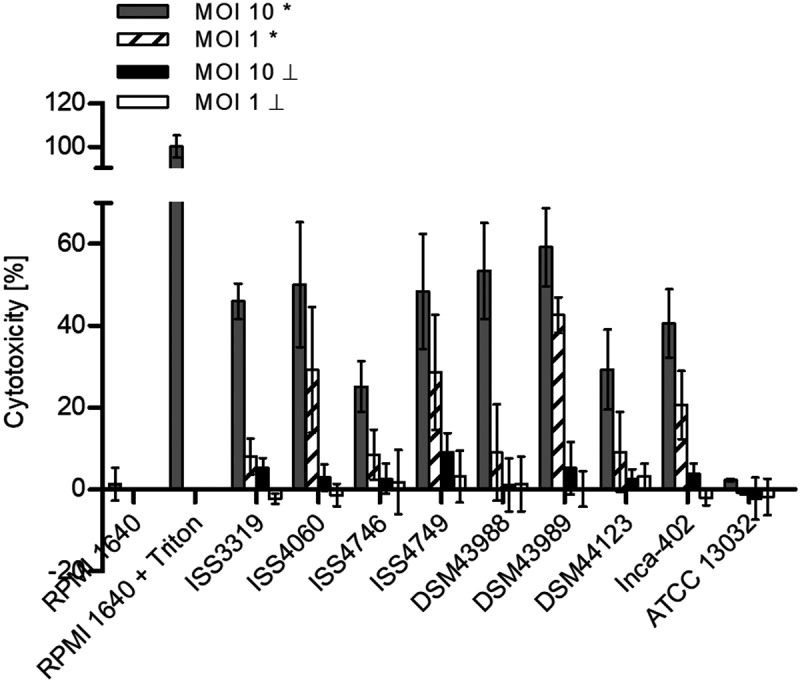
LDH release of cells. The release of lactate dehydrogenase (LDH) as a sign of host cell damage during infection of THP1-Blue NF-KB cells with *C. diphtheriae* was measured using the cytotoxicity detection kit (Roche). Cells were infected for 20 h with viable and UV-killed bacteria of the non-pathogenic strain *C. glutamicum* ATCC 13032 and different pathogenic *C. diphtheriae* wild type strains at an MOI 1 and 10, respectively. Cytotoxicity was calculated based on the absorbance values at 492 and 620 nm. Viable bacteria are indicated with (*) and dead bacteria with (┴). Data shown are mean values of three independent biological replicates each performed in triplicates ± standard deviation.

As mentioned above, the results of NFκB induction in THP-1 cells indicated that some *C. diphtheriae* strains may have detrimental effects on the cells. To test this idea, THP-1 reporter cells were infected for 20 h with the non-pathogenic *C. glutamicum* ATCC 13032 and pathogenic *C. diphtheriae* wild type strains to analyze the release of cytosolic lactate dehydrogenase (LDH) in the supernatant as a sign of host cell damage (Figure 4). In this case, all *C. diphtheriae* strains were able to damage host cells in a dose-dependent manner. The highest cytotoxicity was reached by the toxigenic strain DSM43989 with about 60 and 40% cell death when MOI 10 and MOI 1 of viable bacteria was tested, respectively. This may be most likely caused by diphtheria toxin produced by this strain. The lowest cytotoxic effects of about 25% cell death were detected for the strains ISS4746 and DSM44123 when MOI 10 was used. All other *C. diphtheriae* strains were able to cause at least 40% cell death at MOI 10 and the cytotoxic effect decreased significantly when MOI 1 was applied. As expected, no detrimental effect was observable when UV-killed bacteria, independent of the MOI, were used for infection, indicating that killing of macrophages is an active process conducted by the bacteria. In addition, the non-pathogenic control strain ATCC 13032 did not show any cytotoxic effect on the host cells, independent of the MOI and the physiological condition of the bacteria (Figure 4). In conclusion, decrease of NFκB activation when higher MOIs were used may be a consequence of lower transcription rates due to cell death caused by *C. diphtheriae*. Noteworthy, inducing cell death by *C. diphtheriae* is not only a characteristic of toxigenic strains, but the toxin may probably enhance the cytotoxic effect on the host cell and the diphtheria toxin is not the only mechanism to harm the host cell.

### *Recognition of* C. diphtheriae *by toll-like receptors in human epithelial cells*

**Figure 5.**
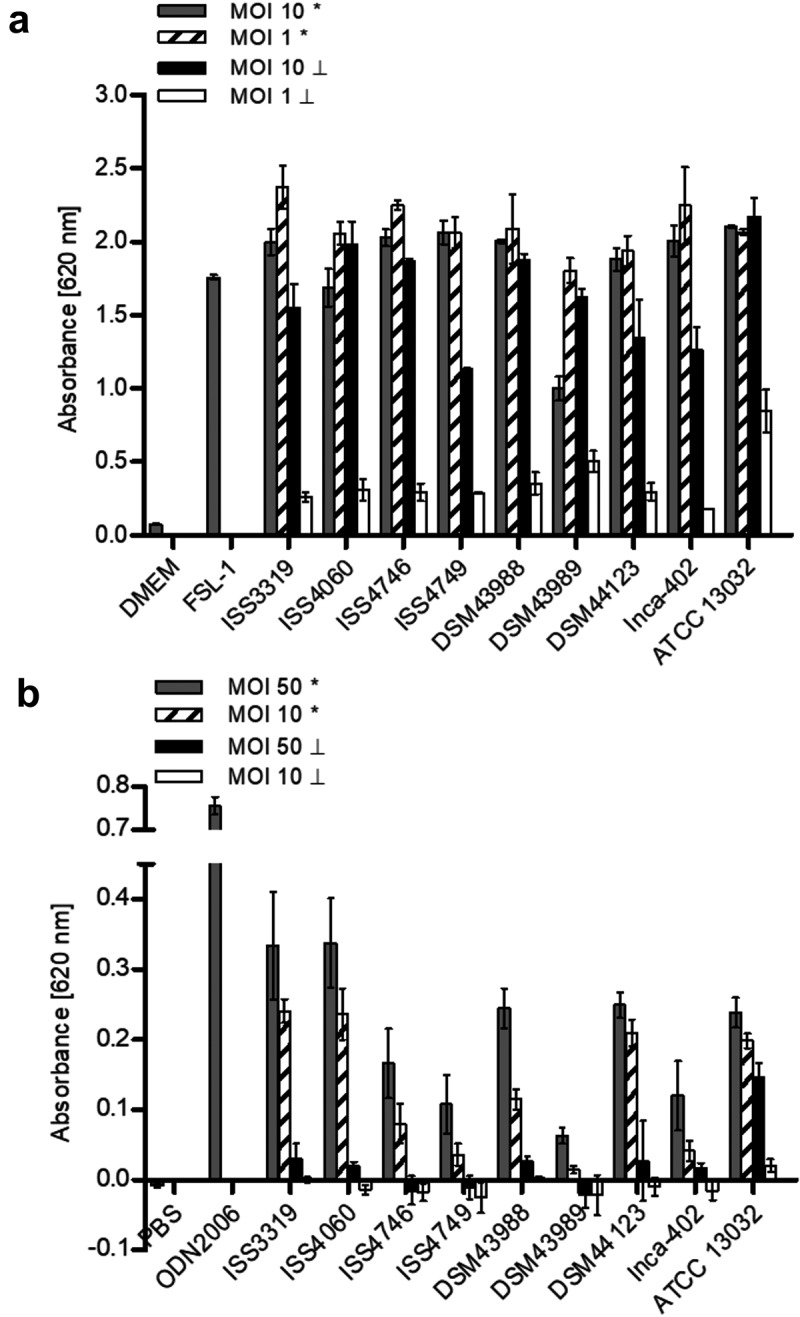
TLR reporter assays. (a) TLR binding of *C. diphtheriae* in HEK-Blue 293 hTLR2 cells. HEK-Blue 293 hTLR2 cells were incubated for 24 h with viable and UV-killed bacteria of pathogenic *C. diphtheriae* and the non-pathogenic *C. glutamicum* ATCC 13032 at an MOI 1 and 10, respectively. Subsequently, supernatants were taken and mixed with QuantiBlue SEAP detection solution leading to color change upon TLR activation. (b) TLR binding of *C. diphtheriae* in HEK-Blue 293 hTLR9 cells. HEK-Blue 293 hTLR9 cells were incubated for 24 h with viable and UV-killed bacteria of pathogenic *C. diphtheriae* and the non-pathogenic *C. glutamicum* ATCC 13032 in HEK-Blue Detection medium at an MOI 10 and 50, respectively. In both cases, TLR binding was detected by measuring absorbance at 620 nm. Viable bacteria are indicated with (*) and dead bacteria with (┴). Data shown are mean values of three independent biological replicates each performed in triplicates ± standard deviation.

Recognition of corynebacterial cell wall glycolipids as well as whole heat-killed bacteria involves TRL2 binding in bone marrow-derived murine macrophages [14]. In order to investigate if TLR2 binding of *C diphtheriae* occurs also in human cells, HEK-Blue 293 hTRL2 were infected for 20 h with seven non-toxigenic strains, one toxigenic *C. diphtheriae* strain and the non-pathogenic *C. glutamicum* ATCC 13032 (Figure 5(a)). MOI 10 and 1 of viable and UV-killed bacteria were tested. As expected, the negative controls water and pure medium showed low absorbance values with less than 0.15. The positive control FSL-1, which is a TLR2 ligand, reached an absorbance value of about 1.75 at 620 nm, while all *C. diphtheriae* strains as well as *C. glutamicum* reached high absorbance values between 1.0 and 2.4 for vital bacteria regardless to the MOI. The toxigenic strain seems to have again a detrimental effect to the cells when an MOI 10 was used, which may be due to the toxin. When UV-killed bacteria are used for infection, the activation of TRL2 seems to be dose-dependent.

### Recognition of intracellular bacteria by TLR9

In order to address the question, if bacteria that show low intracellular CFUs already 2 h post-infection are degraded or not internalized, TLR9 studies with HEK-Blue hTLR9 cells were performed. TLR9 is an intracellular receptor that is located in endolysosomal compartments, where it detects CpG DNA from bacteria and viruses [19]. Therefore, the cells were infected for 24 h with viable and UV-killed bacteria at MOI 10 and 50, respectively (Figure 5(b)). Interestingly, *C. diphtheriae* strains ISS4746, ISS4749 and DSM43989 showed the lowest TLR9 activation. TLR9 activation by *C. diphtheriae* was dose-dependent and dead bacteria showed no significant effect. Moreover, by using this assay, the pathogenicity of the bacteria could be evaluated. The non-pathogenic control strain ATCC 13032 showed very low internalization rates in THP-1 cells (Figure 2(a)), had no detrimental effect to the cells (Figure 4), but revealed high absorbance values in the TLR9 assay (Figure 5(b)). This means that *C. glutamicum* was internalized in high amounts, but much faster degraded than *C. diphtheriae* and was therefore not detectable already after 2 h post-infection. Taken together, these results indicate that strains ISS4746, ISS4749 and the toxigenic strain DSM43989 have unknown mechanisms to avoid to be taken up by the host cell, although they show TLR2 binding and induce inflammatory response in the host.

### Influence of the diphtheria toxin

In all infection studies that have been made thus far with different *C. diphtheriae* wild type strains and epithelial cells [20] or human and murine macrophages in this study, the toxigenic strain DSM43989 was not able to adhere to or to invade into the host cells and it was obviously not taken up in macrophages. Additionally, former studies demonstrated that this strain does not contain mycolic acids in its cell wall [13]. In this study, it was shown that DSM43989 induces inflammatory response in the host as well as host cell damage. The question raised whether the diphtheria toxin or the lack of mycolic acids are responsible for the remarkable behavior of DSM43989. Therefore, we compared the intracellular CFUs of DSM43989 with another diphtheria toxin expressing strain, NCTC13129, in THP-1 cells at 2 and 4 h post-infection (Table 2). Compared to the non-toxigenic strain ISS3319 and the non-pathogenic *C. glutamicum* strain ATCC 13032, both DSM43989 and NCTC13129 are hardly detectable in THP-1 cells at 2 and 4 h post-infection. Since NCTC13129 contains mycolic acids [21] and shows low internalization rates, this result indicates that it may rather be the diphtheria toxin than the structure of the cell wall that influences the infection process.

**Table 2.** Intracellular CFUs [%] of different corynebacteria in THP-1 cells.

Strain	CFUs [%] at 2 h p.i.	CFUs [%] at 4 h p.i.
*C. diphtheriae* ISS3319	2.68 ± 0.34	2.31 ± 0.16
*C. diphtheriae* DSM43989 (*tox^+^*)	0.08 ± 0.02	0.05 ± 0.01
*C. diphtheriae* NCTC13129 (*tox^+^*)	0.10 ± 0.05	0.08 ± 0.01
*C. glutamicum* ATCC 13032	0.25 ± 0.06	0.06 ± 0.05

## Discussion

The innate immune system, an organism’s first line of defense against invading pathogens, works together with the adaptive immune system to maintain physiological homeostasis of the host and to protect it against potentially pathogenic organisms [22,23]. Pattern recognition receptors (PRRs) expressed on the surface of immune cells are crucial for this early detection of invading pathogens as well as to recognize certain endogenous ligands belonging to the family of danger-associated molecular pattern molecules [24,25]. Toll-like receptors (TLRs) are a family of transmembrane receptors, which play a key role in both innate and adaptive immune responses. TLRs and C-type lectin receptors differ in their structures, localization pattern, and the types of ligands they recognize. As effector cells of the innate immune system, macrophages secrete pro-inflammatory and antimicrobial mediators upon phagocytosis of bacteria. The production of IL-6 and G-CSF leads to receptor-mediated activation of the JAK2/STAT3 (Janus Kinase/signal transducer and activator transcription) pathway [26,27], which subsequently leads to the induction of cell survival signals by producing increased levels of the anti-apoptotic proteins Bcl-2 and Bcl-X_L._ In case of *C. diphtheriae*, the knowledge about molecular signaling upon infection of macrophages is scarce. A number of studies showed that *C. diphtheriae*, once considered as strict extracellular pathogen, is able to cause severe invasive diseases, such as osteomyelitis, septic arthritis and endocarditis [6–8], which enhances the compelling need to focus on the infection mechanism of *C. diphtheriae*. In the study presented here, seven non-toxigenic *C. diphtheriae* isolates as well as two toxin-producing strains were characterized with regard to their interaction with BMM and human THP-1 cells. A study conducted by Schick and co-workers on the interaction of heat killed corynebacteria and pure cell wall extracts with the C-type lectin receptor Mincle [14] provided the basis of this study. It was found that Mincle-Fc binds to cell wall extracts of corynebacteria and is required for the release of G-CSF in BMM, and Toll-like receptor 2 (TLR2) is essential for macrophage activation [14]. In order to address the role of Mincle as well as the TLR adaptor protein Myd88 in the infection process of *C. diphtheriae*, in this study, a combination of BMM, Mincle- and MyD88-deficient cells were tested. When phagocytosis of the different *C. diphtheriae* strains was analyzed, no significant difference between BMM and the respective knockout cell line Mincle^−/-^ was observed. In contrast, when Myd88 was absent in BMM almost no intracellular bacteria were detectable. When Mincle was absent in BMM, the G-CSF production was dramatically reduced compared to wild type BMM. This was also the case, when Myd88 was absent. In conclusion, Mincle-binding is crucial for G-CSF production, but not essential for the phagocytosis of the bacteria. Phagocytosis of the bacteria only occurs when the TLR/MyD88 pathway is functional. Furthermore, in this study we could show that viable *C. diphtheriae* interacts with TLR2 of human HEK-Blue 293 hTLR2 cells, which was also the case for heat-killed bacteria and murine macrophages, shown in a previous study [14].

When the interaction of *C. diphtheriae* with THP-1 cells was investigated, the phagocytosis of the bacteria occurred in a strain-specific manner. Remarkably, former studies of Ott and co-workers [20], investigating the infection process of the human epithelial cell lines HeLa and Detroit 562, revealed a very similar infection pattern compared to THP-1 cells in this study. This means that strains that show highest adhesion and invasion rates were also the most phagocytosed in THP-1 cells and *vice versa*. Thereby, it is important to mention, that adhesion to and invasion into epithelial cells is regarded as an active process of the bacteria, while phagocytosis in macrophages is an active process of the host cell. These observations lead to the assumption that some of the *C. diphtheriae* wild type strains are preferentially internalized, while others seem to be avoided by the macrophage. Furthermore, this indicates that the recognition of the bacteria by human cells occurs via the same molecules independent of the cell type.

When NFκB induction in THP-1 cells was analyzed during infection with *C. diphtheriae* a detrimental effect of the bacteria to the cells was assumed, which was confirmed by the LDH release in the supernatant of infected cells, as a sign of host cell damage. Interestingly, the production of reactive nitrogen species in THP-1 cells was contrary to the intracellular CFUs at 2 h post-infection of the different *C. diphtheriae* strains. Due to this observation, the question raised whether the bacteria with low intracellular CFUs are already degraded by NO or are not internalized. The intracellular CFUs at 2 h post-infection were considered as phagocytosis rate thus far, but the bacteria may already be processed at this time point, indicated by high nitric oxide concentrations in infected THP-1 cells that contained almost no viable bacteria. By using TLR9 reporter cell lines, this question was addressed. Since TRL9 is an endolysosomal receptor, that binds CpG unmethylated bacterial and viral DNA, it became evident that bacteria that show low intracellular CFUs were not or weakly detected by TLR9, indicating that these strains were not endocytosed by the cell.

In conclusion, comparison of murine (BMM) and human phagocytes (THP-1) revealed similar intracellular CFUs for the different *C. diphtheriae* strains. TLR 9 reporter assays confirmed that *C. diphtheriae* that showed low intracellular CFUs were not detected by TLR9 in the endosome indicating that these strains were not taken up by the cell. In contrast, the non-pathogenic control *C. glutamicum* ATCC 13032 showing also almost no intracellular CFUs independent of the cell line, was recognized by TLR9, indicating that the bacteria are taken up by the cell and degraded immediately after endocytosis. In terms of G-CSF and IL-6 production, no significant differences between BMM and THP-1 for *C. diphtheriae* were observed, while the non-pathogenic *C. glutamicum* induced only half of the cytokine secretion in response to infection independent of the host cell. By using murine Mincle- and Myd88-deficient cells, it could be shown that the absence of Mincle resulted in reduced G-CSF production, while this had no influence on the uptake of the bacteria in comparison to BMM. In contrast, when MyD88 was absent, both the uptake of the bacteria as well as IL-6 and G-CSF production were blocked. Consequently, phagocytosis of the bacteria only occurs when the TLR/MyD88 pathway is functional, which was also confirmed by showing that all corynebacteria used in this study were able to interact with human TLR2.

However, it was not clear thus far, why the toxigenic strain DSM43989 showed very low adhesion to and invasion rates into Detroit 562 and HeLa cells and almost no intracellular CFUs in human macrophages [20]. A putative reason may be the lack of mycolic acids, which may consequently` lead to an impaired interaction of the bacteria by the host cell. The comparison with another toxigenic *C. diphtheriae* strain NCTC13129, that contains mycolic acids in its cell wall, revealed similar intracellular CFUs in THP-1 cells. These results indicate that the low number of intracellular CFUs of toxigenic strains is most likely not connected to the presence or absence of mycolic acids. Although the influence of the diphtheria toxin on the infection process of these strains may explain the cytotoxic effect of DSM43989 to THP-1 cells, this does not explain the low uptake by murine phagocytes, since murine cells are not susceptible to the diphtheria toxin [28]. To solve this question, future experiments have to be carried out concerning the susceptibility of murine cells toward the diphtheria toxin.

## Summary

**Figure 6.**
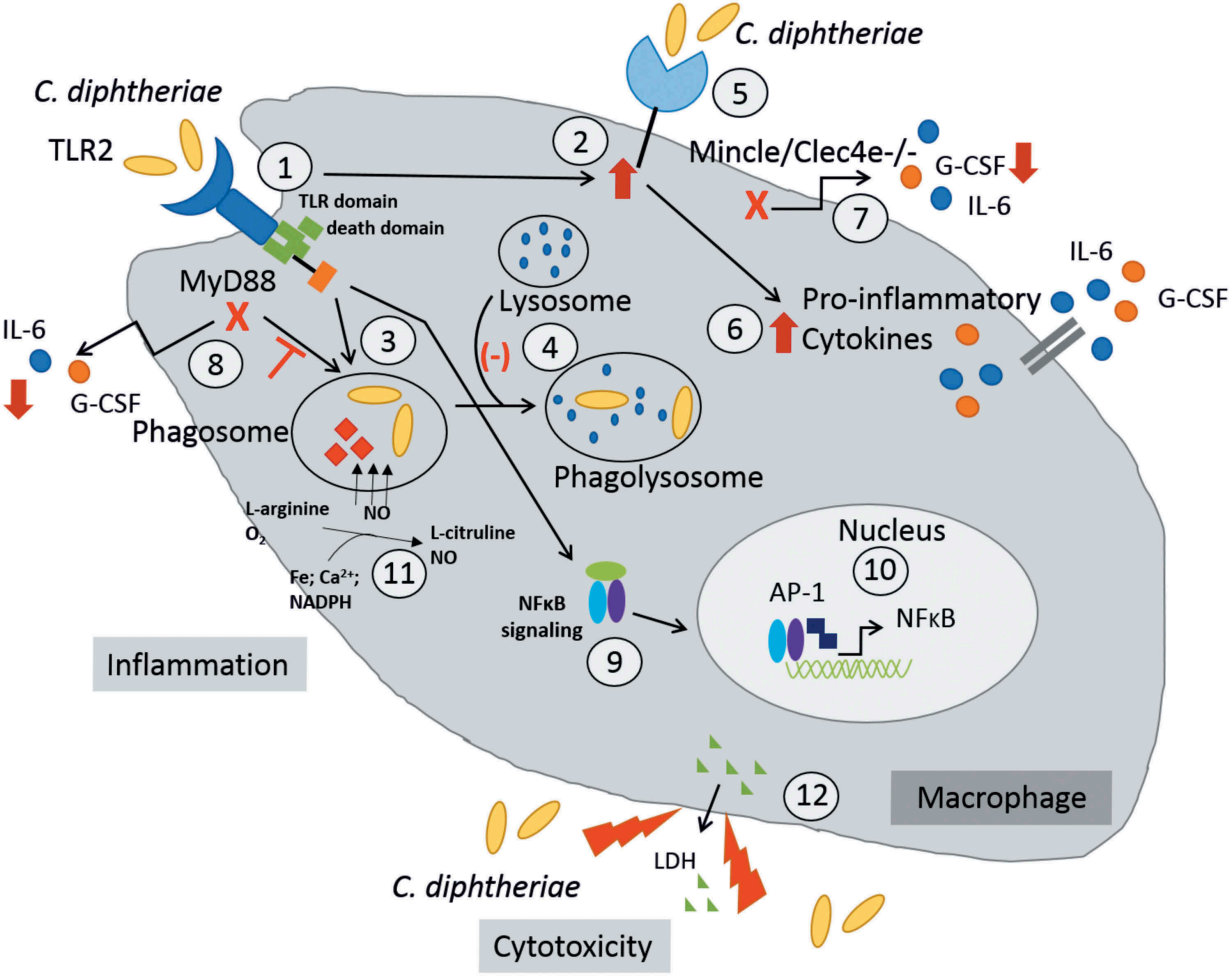
*C. diphtheriae* recognition by macrophages. Binding of *C. diphtheriae* by TLR2 (1) leads on the one hand to upregulation of the C-type lectin receptor Mincle (2) and on the other hand, to phagocytosis of the bacteria (3), resulting in phagosome-lysosome fusion, which is somehow delayed by *C. diphtheriae* (4). Furthermore, binding of *C. diphtheriae* to Mincle (5) triggers the production of pro-inflammatory cytokines (6), which was confirmed by reduced cytokine production in Clec4e^−/-^ cells (7). Additionally, in Myd88-deficient cells the cytokine production as well as the uptake of the bacteria was completely blocked (8). Further signs of inflammation caused by pathogenic corynebacteria are the activation of NFκB-signaling (9), resulting in upregulation of pro-inflammatory genes (10), and the production of nitric oxide (NO) (11). In case of the infection of THP-1 cells, a cytotoxic effect of *C. diphtheriae* was detectable by LDH release (12).

In contrast to the non-pathogenic strain *C. glutamicum* ATCC 13032, *C. diphtheriae* is able to cause host immune response by inducing inflammatory cytokine and NO production, is able to delay phagolysosome formation and has a cytotoxic effect on the cells (Figure 6). The TLR2/Myd88 pathway is required for phagocytosis of *C. diphtheriae* and leads to NFκB-signaling as well as upregulation of the C-type lectin receptor Mincle. Since the detected intracellular CFUs are strain-specific and/or cell line-specific, the bacterial antigen, which binds to the host cell receptor, may be expressed in various amounts across the different *C. diphtheriae* strains. On the one hand, these results emphasized the high virulence potential of *C. diphtheriae* by activating the innate immune system and causing inflammatory response in phagocytic cells. On the other hand, since both *C. diphtheriae* and *C. glutamicum* bind obviously to TLR2 and Mincle, *C. diphtheriae* may express further proteins/molecules interacting with host cells, enabling the bacteria to trigger host immune response. Based on the results carried out in this study, our future work will be focused on detailed elucidation of inflammatory pathways triggered by *C. diphtheriae* as well as the identification of the bacterial proteins that are recognized by the host cell.

## References

[1] Lehmann KB, Neumann R. Atlas und Grundriss der Bakteriologie und Lehrbuch der speziellen bakteriologischen Diagnostik. Munich: Lehmann; 1896.

[2] Barksdale L *Corynebacterium diphtheriae* and its relatives. Bacteriol Rev. 1970;34:378–422. 4322195 10.1128/br.34.4.378-422.1970 PMC378364

[3] Burkovski A Diphtheria and its etiological agents In: Burkovski A, editor. *Corynebacterium diphtheriae* and related toxigenic species. Dordrecht: Springer; 2014 p. 1–14.

[4] Sangal V, Hoskisson PA Corynephages: infections of the infectors In: Burkovski A, editor. *Corynebacterium diphtheriae* and related toxigenic species. Dordrecht: Springer; 2014 p. 67–81.

[5] Hirata R Jr., Pereira GA, Filardy AA, et al Potential pathogenic role of aggregative -adhering *Corynebacterium diphtheriae* of different clonal groups in endocarditis. Braz J Med Biol Res. 2008;41:986–991. 19099151 10.1590/s0100-879x2008001100007

[6] Puliti M, von Hunolstein C, Marangi M, et al Experimental model of infection with non-toxigenic strains of *Corynebacterium diphtheriae* and development of septic arthritis. J Med Microbiol. 2006;55:229–235. 16434717 10.1099/jmm.0.46135-0

[7] Peixoto RS, Pereira GA, Sanches Dos Santos L, et al Invasion of endothelial cells and arthritogenic potential of endocarditis-associated *Corynebacterium diphtheriae*. Microbiology. 2014;160:537–546. 24344208 10.1099/mic.0.069948-0

[8] Peixoto RS, Hacker E, Antunes CA, et al Pathogenic properties of a *Corynebacterium diphtheriae* strain isolated from a case of osteomyelitis. J Med Microbiol. 2016;65:1311–1321. 27902402 10.1099/jmm.0.000362

[9] Ishikawa E, Ishikawa T, Morita YS, et al Direct recognition of the mycobacterial glycolipid, trehalose dimycolate, by C-type lectin Mincle. J Exp Med. 2009;206:2879–2888. 20008526 10.1084/jem.20091750 PMC2806462

[10] Werninghaus K, Babiak A, Gross O, et al Adjuvanticity of a synthetic cord factor analogue for subunit *Mycobacterium tuberculosis* vaccination requires FcRgamma-Syk-Card9 dependent innate immune activation. J Exp Med. 2009;206:89–97. 19139169 10.1084/jem.20081445 PMC2626670

[11] Schoenen H, Bodendorfer B, Hitchens K, et al Cutting edge: mincle is essential for recognition and adjuvanticity of the mycobacterial cord factor and its synthetic analog trehalose-dibehenate. J Immunol. 2010;184:2756–2760. 20164423 10.4049/jimmunol.0904013 PMC3442336

[12] Deguine J, Barton GM MyD88: a central player in innate immune signaling. F1000Prime Rep. 2014;6:97. 25580251 10.12703/P6-97 PMC4229726

[13] Ott L, Hacker E, Kunert T, et al Analysis of *Corynebacterium diphtheriae* macrophage interaction: dispensability of corynomycolic acids for inhibition of phagolysosome maturation and identification of a new gene involved in synthesis of the corynomycolic acid layer. PLoS ONE. 2017;12:e0180105. 28686600 10.1371/journal.pone.0180105 PMC5501465

[14] Schick J, Etschel P, Bailo R, et al Toll-like receptor 2 and Mincle cooperatively sense corynebacterial cell wall glycolipids. Infect Immun. 2017;85:e00075–17. 28483856 10.1128/IAI.00075-17 PMC5478951

[15] Lang R, Rutschman RL, Greaves DR, et al Autocrine deactivation of macrophages in transgenic mice constitutively overexpressing IL-10 under control of the human CD68 promoter. J Immunol. 2002;168:3402–3411. 11907098 10.4049/jimmunol.168.7.3402

[16] Kawai T, Adachi O, Ogawa T, et al Unresponsiveness of MyD88-deficient mice to endotoxin. Immunity. 1999;11:115–122. 10435584 10.1016/s1074-7613(00)80086-2

[17] O’Neill LA, Bowie AG The family of five: TIR-domain containing adaptors in Toll-like receptor signaling. Nat Rev Immunol. 2007;7:353–364. 17457343 10.1038/nri2079

[18] Tripathi P, Tripathi P, Kashyap L, et al The role of nitric oxide in inflammatory reactions. FEMS Immunol Med Microbiol. 2007;51:443–452. 17903207 10.1111/j.1574-695X.2007.00329.x

[19] Hemmi H, Takeuchi O, Kawai T, et al A Toll-like receptor recognizes bacterial DNA. Nature. 2000;408:740–745. 11130078 10.1038/35047123

[20] Ott L, Scholz B, Hoeller M, et al Induction of the NFκ-B signal transduction pathway in response to *Corynebacterium diphtheriae* infection. Microbiology. 2013;159:126–135. 23125120 10.1099/mic.0.061879-0

[21] Cerdeño-Tárraga AM, Efstratiou A, Dover LG, et al The complete genome sequence and analysis of *Corynebacterium diphtheriae* NCTC13129. Nucleic Acids Res. 2003;31:6516–6523. 14602910 10.1093/nar/gkg874 PMC275568

[22] Kulkarni OP, Lichtnekert J, Anders HJ, et al The immune system in tissue environments regaining homeostasis after injury: is “inflammation” always inflammation? Mediators Inflamm. 2016;2016:2856213. 27597803 10.1155/2016/2856213 PMC4997018

[23] Medzhitov R, Janeway CA Jr. Decoding the patterns of self and nonself by the innate immune system. Science. 2002;296:298–300. 11951031 10.1126/science.1068883

[24] Shcheblyakov DV, Logunov DY, Tukhvatulin AI, et al Toll-like receptors (TLRs): the role in tumor progression. Acta Naturae. 2010;2:21–29. 22649649 PMC3347570

[25] Takeuchi O, Akira S Pattern recognition receptors and inflammation. Cell. 2010;140:805–820. 20303872 10.1016/j.cell.2010.01.022

[26] Hirano T, Ishihara K, Hibi M Roles of STAT3 in mediating the cell growth, differentiation and survival signals relayed through the IL-6 family of cytokine receptors. Oncogene. 2000;19:2548–2556. 10851053 10.1038/sj.onc.1203551

[27] Solaroglu I, Cahill J, Jadhav V, et al A novel neuroprotectant granulocyte-colony stimulating factor. Stroke. 2006;37:1123–1128. 16514095 10.1161/01.STR.0000208205.26253.96

[28] Mitamura T, Higashiyama S, Taniguchi N, et al Diphtheria toxin binds to the epidermal growth factor (EGF)-like domain of human heparin-binding EGF-like growth factor/diphtheria toxin receptor and inhibits specifically its mitogenic activity. J Biol Chem. 1995;270:1015–1019. 7836353 10.1074/jbc.270.3.1015

[29] Bertuccini L, Baldassarri L, von Hunolstein C Internalization of non-toxigenic *Coryne-bacterium diphtheriae* by cultured human respiratory epithelial cells. Microb Pathog. 2004;37:111–118. 15351033 10.1016/j.micpath.2004.06.002

[30] Nakao H, Pruckler JM, Mazurova IZ, et al Heterogeneity of diphtheria toxin gene, *tox*, and its regulatory element, *dtxR*, in *Corynebacterium diphtheriae* strains causing epidemic diphtheria in Russia and Ukraine. J Clin Microbiol. 1996;34:1711–1716. 8784575 10.1128/jcm.34.7.1711-1716.1996 PMC229100

[31] Trost E, Ott L, Schneider J, et al The complete genome sequence of *Corynebacterium pseudotuberculosis* FRC41 isolated from a 12-year-old girl with necrotizing lymphadenitis reveals insights into gene-regulatory networks contributing to virulence. BMC Genomics. 2010;11:728. 21192786 10.1186/1471-2164-11-728 PMC3022926

[32] Public Health Laboratory Service Diphtheria acquired during a cruise in the Baltic Sea. Commun Dis Rep CDR Wkly. 1997;7:207. 9198300

[33] Abe S, Takayama K, Kinoshita S Taxonomical studies on glutamic acid producing bacteria. J Gen Microbiol. 1967;13:279–301.

[34] Tsuchiya S, Yamabe M, Yamaguchi Y, et al Establishment and characterization of a human acute monocytic leukemia cell line (THP-1). Int J Cancer. 1980;26:171–176. 6970727 10.1002/ijc.2910260208

