# Supplementary figures and images for: Of mice and men: Interaction of Corynebacterium diphtheriae strains with murine and human phagocytes
Source: Virulence. 2019 May 6;10(1):414–28. doi: 10.1080/21505594.2019.1614384 (PMC6527023; doi:10.1080/21505594.2019.1614384)

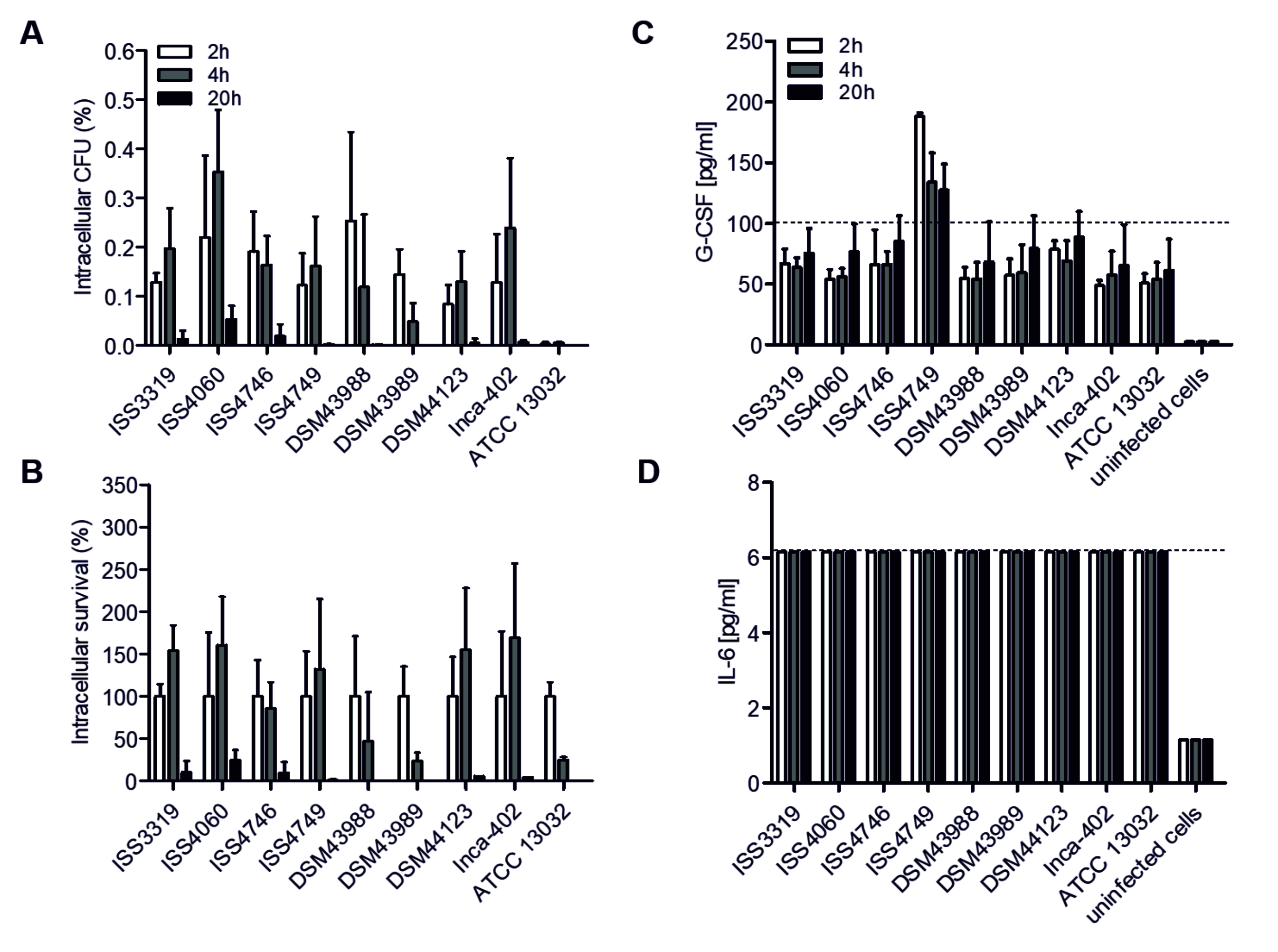

Supplement: Supplemental Material [file kvir-10-01-1614384-s001.jpg]

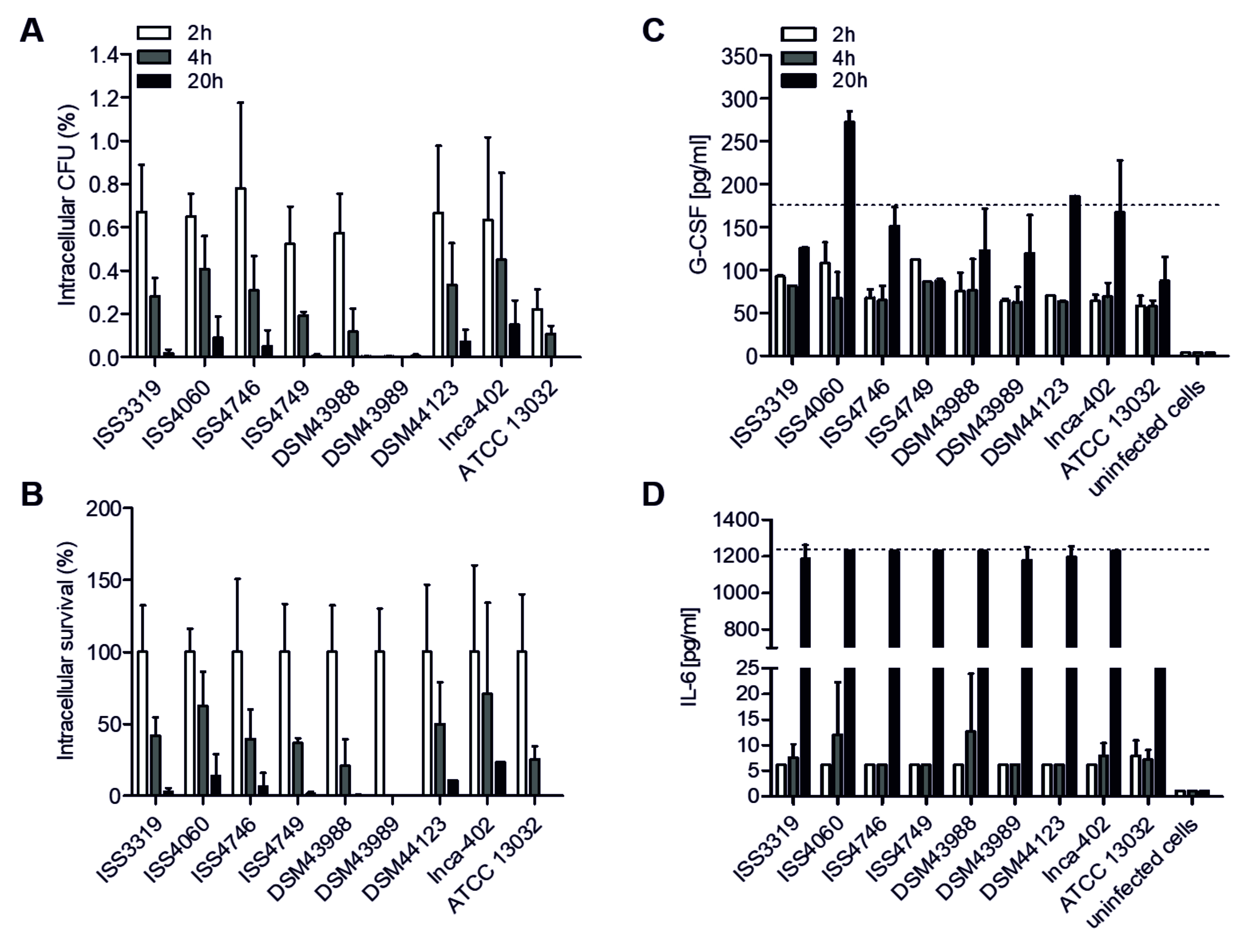

Supplement: Supplemental Material [file kvir-10-01-1614384-s002.jpg]

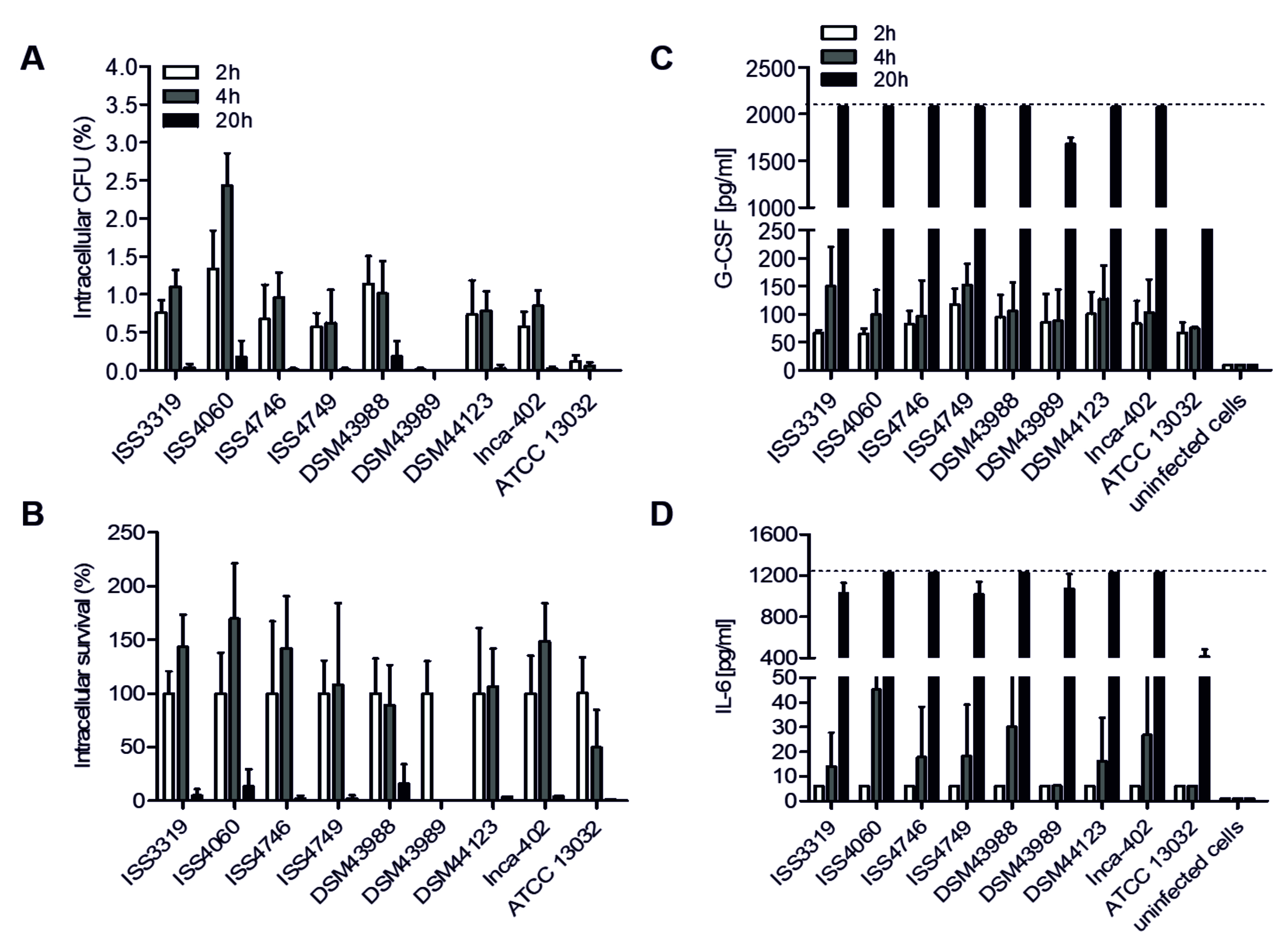

Supplement: Supplemental Material [file kvir-10-01-1614384-s003.jpg]
